# The Development of Dentine

**Published:** 1877-11

**Authors:** C. Sihler

**Affiliations:** Fellow in Biology, Johns Hopkins University, Baltimore, Md.


### ARTICLE II.

### *The Development of Dentine.*

BY C. SIHLER, M. D.

Fellow in Biology, Johns Hopkins University, Baltimore, Md.

The view then at which I have arrived, after investigating the dead and living dentine, as to the structure of this tissue is 1st, the essential parts of the living and growing dentine, the oval elements or pink bodies attached to the opening with their nuclei of the dentinal canals, and in direct connection with the dentine. 2nd. There can be recognized in this tissue the dentine, as can elsewhere the germinal matter or bioplasm and the formed matter. The former has been pointed out by the staining, and has been shown that it exists in the dentinal canals, the odontoblasts and their nuclei. The latter, *i. e.*, the formed matter we have shown to consist of two kinds of substances, by the aid of muriatic acid: First, a homogeneous one in which the lime-salts are infiltrated, a second one, of the nature of yellow elastic tissue substance, making up the walls of the dentinal tubules, and being in continuity with the odontoblasts. That it is prolonged into (or on) bioblast, we have shown by isolating an odontoblast while in connection with the dentinal tubules, and that this process of the odontoblast is not altogether germinal matter, or merely a semi-fluid soft mass; can be proved by the stretching it will permit. Where the germinal matter ends, and the formed matter begins, is just as difficult at times to say, as where day ends and night begins, for one must always bear in mind in using this term, that the distinction made between forming substance or bioplasm, or germinal matter, and formed matter is a termination not made according to the idea (category) of form and appearance or locality, but according to the idea of force or substance. Hence, we may have particles of formed matter and particles of germinal matter mixed up. The body of the odontoblast and the material in the dentinal tubules, seem to me to be of this nature, *i. e.*, mixed, while the nucleus seems to me to be active bioplasm. In making histological investigations, one must not forget that one is interrupting nature and interfering with processes. I look upon the odontoblast as though here the germinal matter was being converted into formed matter, and was being separated into the elastic material, which when condensed, will make up the walls of the tubes and side tubes,—and into the homogeneous glue-like substance making the general matrix of the dentine, and I look upon the contents of the dentinal tubes which can be stained, as upon germinal matter where change into formed matter is slowly going on, while the nucleus, I consider, as the agent which converts pabulum or food into germinal matter.

This view in the structure of dentine will agree with that of Beale, if Beale will admit that there is a special wall to the dentinal canal, which is a direct production of the germinal matter, as the fibre of the ligamentum nuchae is a production of the bioplasts attached to them, and which is made *pari parsu* with the general glue-like matrix of the dentine.

This view will agree with that of Salter, if Salter will call his "thick fluid" in the dentinal canals germinal matter, and admit that he does not prove the calcification of the dentinal tubes.

This view will agree with that of Waldeyer, if Waldeyer admits that his dentinal sheaths and the fibres which he describes as possessing a remarkable elasticity, are one and the same thing.

This view will agree with that of Koelliker, if Koelliker admits that his dentinal tubules (Neumann's sheaths) and his processes of the odontoblasts are one and the same thing.

This view agrees with that of Tomes, if Tomes will admit that he fails to prove that there is a dentinal tubule, and admit further that his soft fibril is one and the same thing with the dentinal tubule.

These three views will agree with mine, if they will allow me to call the contents or inner portion of their soft fibril bioplasm—if they allow me to call the outer portion of their fibril the dentinal tubule, and the material which according to their own statements can be stretched so much,—yellow elastic tissue substance.

But I will quote the authors themselves, and try to point out where and why I am not convinced by their arguments. Tomes says: "I had, however, but little expectation of finding that one of the most important points in dental structure had been overlooked, namely that each dentinal tube is permanently tenated by a soft fibril, which after passing from the pulp into the tube follows its ramifications. With proper care in manipulating, nothing is more easy than to demonstrate the existence of the dentinal fibres in any tooth which has recently been extracted. If a thin section be made in a plane parallel with the direction of the tubes, and the sections be afterward torn in a direction transverse to that of the tubes, many of the fibrils will be seen projecting from the torn edges. (Fig. 124.) It is desirable in repeating the experiment to place the decalcified section upon a slide before tearing, as in moving it from the surface upon which it has been torn, some of the longer fibrils may be folded back upon the body of the specimen, and thus become obscured from view. Where the separation between the torn surfaces has been but slight, we may often see a fibril unbroken stretching across the interval, which separates the orifices of the tubes to which it belongs.

In order to demonstrate the connection of the fibrils with the pulp, fine sections should be made with a sharp knife from the edge of the pulp cavity. In this manner I obtained the specimen from which Mr. DeMorgan has been kind enough to draw the accompanying illustration, showing the fibrils stretching from the pulps to the displaced dentine, and some of them passing out on the other side of the fragment. That the fibrils proceed from the pulp may be seen by carefully fracturing a fresh tooth with as little displacement of the fractured parts as possible, and then by slowly removing the pulp from its place in the tooth, we shall be enabled to examine the fibrils which have been drawn out from the tubes. By this procedure some of the fibrils will be withdrawn from their normal position in the dentine in the greater part of their length, a few of them retaining short lengths of their branches, but sufficiently long to show that they have come from the branches of the dentinal tube."

These same arguments it will be noticed we have used above in proving the presence of the dentinal tubule, and have found the same appearance in dead teeth. We have seen when describing the nature of the dead and dry dentine, that a tubule and a fibril cannot be distinguished one from another by merely being seen, especially along their sides. Further we have shown that by decalcifying, the fibrils apparently drawn out from the dentinal canal (I do not say tubes) are identical with the tubes themselves, and all the phenomena described by Tomes can be produced by tubules as well as by fibrils.

Tomes quotes Koelliker, who gives facts supporting our view, but I fail to see that Tomes either sees the point or refutes it. He quotes the following, viz:

"Prof. Koelliker in his account of the development of dentine, describes and figures processes extending from the peripheral cells of the dentinal pulp in developing teeth, but he does not recognize the tube fibril; indeed he, as before cited describes the tubes as filled with fluid. Mr. Lent in a paper published in 1855, gives a similar description to that published by Mr. Koelliker, and says that the cell fibres are but seen in teeth which are but little advanced in development. Mr. Huxley states that in a solitary instance he observed a fibre pass a short distance into the dentine."

"Both, Mr. Koelliker and Lent, regard the processes which they observed extending from the peripheral cells of the pulp in forming teeth, as organisms for the development of the dentinal tubes. The latter author, near the conclusion of his article on the development of dentine, states that the processes of the cells are the dentinal tubes. He observes further on that the fact first observed by Mueller and then by Koelliker, that the dentinal tubules possess separate walls, which can readily be isolated and explained by the history of the development—the wall of the dentinal canal is identical with the cellular membrane of the ivory cell."

If any one else understands why Tomes quotes Koelliker, he accomplishes more than I can. To me it appears that this quotation might have shown to Tomes that it is impossible to recognize his soft fibril, that the arguments given to support it, will support Koelliker's and Lent's views a good deal better than his own.

Salter takes the same views of Tomes' soft fibrils. We can in giving his criticism at the same time get Salter's view of the nature of dentine. He says, viz:

"Dentine or ivory, which constitutes the bulk of the tooth is a tissue of bony hardness; it consists of earthy matter to the extent of not less than three fourths of its weight. Histologically it is composed of a series of minute tubes about 1-9000 of an inch in diameter, radiating from the central hollow chamber or pulp cavity in which is lodged the soft vascular organ the pulp, which is mainly concerned in the nutrition of the teeth. Between the dentinal tubes is a hyaline structure called the intertubular tissue. The tubes pursue a wavy course and branch, more or less especially in the crowns of the teeth near the surface, and occasionally they exhibit dilatations somewhat like bone lacunæ.

The dentinal tubes have extremely thin walls, which can scarcely be said to be visible in the hard tissues when seen in profile, but which in transverse section as viewed by high powers of the microscope display a broad brilliant ring of considerable thickness around each. The appearance is an optical illusion, and has given rise to much error in interpreting the histological elements of dentine. Whether the broad ring is the result of the curious phenomenon of irradiation or the diffraction of light by the thin cut edge of the tube, I am not prepared to say. But the appearance is not without parallels, which are thus explained. This fact was first pointed out by Henle, in 1841, and is easy of confirmation. It can be demonstrated both by analysis and by synthesis. In some fractures of sections of dentine, their fine tubes are seen standing out rigid and calcified from the broken edge. The animal basis of the dentinal tubes and that of the intertubular substance are different, the former is considerably denser, and after decalcification strong hydrochloric acid will remove the intertubular substance, or render it absolutely transparent—thus the tubes can be isolated, and their minute diameter corresponding with the dark dentinal tube, as seen in profile view of hard sections is at once recognized. If tubes thus distinctively free from all surrounding walls be traced along their course, occasionally some are seen turning their broken edge towards the observer when the rings in question are apparent and the illusion is manifest.

Synthetically this question of histology is demonstrated with equal clearness. In studying the development of dentine it is found that upon the formative pulp, there is arranged a series of columnar cells, constituting the membrane eboris, from the dentinal extremity of which minute tubular threads project, and as the dentine forms from without inwards, these are prolonged centripetally, a homogeneous blastema separating them, and being calcified with them. No other elements hitherto discovered enter into the formation of the dentine, and the minute tubes freed by the decalcification and the solution of the intertubular tissue in dentine, through the action of hydrochloric acid are identical with the thread like prolongation seen on the ends of the columnar cells of the dentinal pulp. These minute tubules of dentine when free and soft, have a certain resemblance to nerve fibres, and this circumstance combined with the belief that the luminous rings around the tubes represent the walls of comparatively large hollow canals, has led a distinguished microscopist to conclude that they are nerves occupying the cavities of such canals, and unaware that histological facts had been recorded both in description and illustration by Henle long previously, he presented to the Royal Society a memoir on the presence of fibrils of soft tissue in the dentinal tubes, " and in that memoir he speaks of them as organs of sensation. But the truth is that they are the dentinal tubes themselves, and there is nothing whatever separating the bodies from the intertubular substance. In the observations made by this author, great stress was laid upon the examinations being made on perfectly fresh specimens, so that the soft structures may not have suffered injury or decay. The minute tubules may, however, be demonstrated just as well in the oldest specimen of dentine. The accompanying illustration is from a specimen prepared by myself of a portion of decalcified dentine from the tooth of an ancient Briton, that had been entombed probably for not less than 2,000 years, all soft uncalcified tissues must have perished for ages."

"The dentinal tubes are hollow and appear to contain a dense fluid plasma, and further on, the axis of the tube therefore, whatever may be its disputed nature being occupied by a fluid."

So far Salter, and I will say that I agree with his arguments against the views of Tomes, but have to remonstrate as regards a few statements. I am by no means convinced that the dentinal tubule is calcified; there is no proof given, and from the very nature of the material of which the elastic tissue is composed, I think it must be uncalcified. As far as 2,000 years are concerned, time is nothing, and we know that muriatic acid speedily dissolves the homogeneous matrix and for quite a time seems powerless against the tubes, and further the very existence of a tooth speaks for the possibility of the presence of the yellow elastic, which can stand certainly more acid and alkalies than the glue or intertubular matrix of dentine, and if it can stand more of chemical action, why not more time? The limesalts are not such powerful preservers. I must object to simply calling the contents of the tubes fluid, dense plasma and to saying after describing the tubes and intertubular substances, "no other element enters, etc.," that is reasoning just as if one did not recognize the nucleus in a liver cell, or the connective tissue corpuscle in fibrous tissue, or the nuclei in muscle and nerve. As far as the description of the formation of dentine is concerned it is good enough as far as it goes, but does not mention the formation of the side tubes.

But Tomes gives some other facts, some of which Salter also quotes which are of importance; he says, if a fibril be examined in its natural condition by the aid of a $\frac{1}{8}$ object glass, it will be found to consist of an almost structureless tissue, transparent and of a comparatively low refractive power. In glycerine the fibrils are scarcely visible. At present it admits of doubt, whether they are tubular or solid. In some cases there is an appearance of tubularity, but being cylindrical this may be a mere optical effect. When accidentally stretched between two fragments of dentine, the diameter of the fibril becomes much diminished, and when broken across, a minute globule of transparent but dense fluid may sometimes be seen at the broken end gathered into a more or less spherical form. These appearances may be explained by assuming that the fibril consists of a sheath, containing a semi-fluid matter similar to the white fibrillæ of nerves, etc."

It is somewhat surprising to see Tomes calling a thing which can be stretched so much as it can, and of diameter which is diminished by stretching, and which seems to contain a dense fluid,—a soft fibril. The trouble with Tomes' description of dentine is, that at first he assumes the dentinal tubes, then, when he comes across them in reality, he takes them for something else, namely, fibrils. I think it will be noticed that his dense fluid in his soft fibrils corresponds to the germinal matter in the tubes, his soft fibrils, or more accurately the outer dense portions of his fibril, are our dentinal tubes.

Koelliker, who if I understand him rightly, had entertained similar views as Salter on dentine, has changed his views in the last edition of his work on histology, and on page 336 says, " although I had been the first one who had isolated the dentinal tubules to a great extent, I was afterwards misled, after Tomes had described a soft fibril in every dentinal tubule, to take the tubes which I had produced and those fibrils for one and the same thing, against which identification Neumann raised objections. He showed in a most excellent little work that the dentinal tubules had special calcified walls (dentinal sheaths Neumann,) and that these contained in their interior a soft fibril. (Tooth fibril, fibre of Tomes.) Although recently H. Herbs has adopted my latter views, I cannot but think that Neumann is in the right. The arguments are: 1. " If by boiling in caustic potash of sections of teeth, or by prolonged putrid maceration of whole teeth, the soft parts of the teeth are destroyed, the walls of the dentinal canals can after the removal of the limesalts be brought into view by aid of the measures which I mentioned, while the fibrils can in no way be shown."

Here I would ask: 1. How can one know at what time all the soft parts are destroyed? 2. What is really meant by soft parts? 3. Cannot some uncalcified substances stand as much chemical action and time as some calcified ones?

I have found that when I treated my sections with caustic soda until I thought that all the soft parts were destroyed, that the whole section melted down before the hydrochloric acid like ice in warm water, and as far as putrefaction is concerned, that is a little doubtful manœuvre, for in decaying teeth, where we are sure that putrefaction is really at work, we see that the whole of the dentine sheaths and all is destroyed. The very existence of a tooth proves that putrefaction has not been going on very intensively. If one exposes, for example, the pulp and pericementum to to caustic soda, and boiling at that, or too strong muriatic acid he will find that these "soft" tissues although soft in a certain sense have properties which make them persistent.

The second argument of Koelliker to prove his statement:

2. "The elements isolated by Neumann and myself can, as I have said from the beginning, plainly be distinguished as tubules.

I have no doubt at all that these elements are tubules, but I cannot see that their calcification is in any way proved by the arguments so far.

3. Upon the examination of dentine which is undergoing formation it is found that every forming cell (odontoblast) sends a soft fibril into the interior of the dental tubule (Lent, I. Neumann) and there can (Neumann) be shown with the aid of muriatic acid besides these fibres also the dentinal sheaths.

Here I would again like to ask, why Koelliker quotes Lent, Neumann and himself, in proving the existence of the fibril as a process of the odontoblast, and Neumann only in proving the existence of the sheath aside of or with the fibre?

Further this argument is nothing further than a repetition of the proposition to be first proven, and every one has of course a right to disbelieve this statement, until the exact method is given how such facts can be obtained. I have made a section through the root of a developing tooth so very thin that there was to be seen but one layer of tubes as it were, and in front of a small number of canals the odontoblasts were still attached, while in the greater portion they were detached. This section I exposed to the action of strong muriatic acid; after this had operated long enough to dissolve all the homogeneous matrix, I found as many fibres apparently as there were canals before, and where there had been an odontoblast this was connected with a fibre or tube. Of course where there had been no odontoblast attached, there the fibre or tube ended where the dentine had ended before. Now where are the sheaths of Neumann, together with the fibril of Tomes? The process of the odontoblasts are either the one or the other, or are we to call those where the odontoblast is detached the sheaths of Neumann, and where it is not detached the fibre of Tomes, so that all the investigators may have the honor of some discovery.

Of course with carmine one can prove that within the tubule or within the fibre, or in the interior of the fibre there is another kind of substance, and if to that, without the covering the term soft fibril will be given, I will not object, although it seems an unfelicitous term. But terms I intend not to fight, but to mix up several things and to create two things out of one, that mode of reasoning I cannot agree to.

4. "The same cell processes can also be seen in fully developed teeth proceeding from the cells on the surface of the pulp and entering the dentinal canals, and in longitudinal and cross-sections of decalcified dentine the soft fibres can be recognized in situ."

As far as this evidence is concerned it is not at all convincing to me, for decalcification will disturb the structure enough to make observations upon the contents of the dentinal canals a little doubtful, as far as seeing a soft fibril in longitudinal sections, I fail to see how one can see the softness, and how one can fail to see something like a fibre; and as far as cross-sections are concerned, I suppose in fresh teeth of course there is within the tubule, or sheath, or fibre the material which is stained with carmine, and which is pressed out from the fibril according to Tomes in round drops, and a cross-section will of course not allow an empty tube to appear. But I admit the tube, and I admit its contents, but only cannot see how the sheath of Neumann and the fibril of Tomes can be shown or seen side by side.

5. "On picking to pieces sections of decalcified dentine, fibrils can frequently be seen protruding along the edges, (Tomes) but it is to be taken into consideration that also the dental sheaths can be seen protruding in this way."

Here Koelliker admits himself the possibility of taking a tubule for a fibre, and *vice versa*, and it is not necessary to say any more.

Foey and Waldeyer have about the same view on dentine as Tomes and Koelliker, and the same arguments hold good against them as those against Tomes and Koelliker, but for completeness-sake we will quote Waldeyer also. He says (Strickers' Manual,) page—— "The dentinal fibres constitute the soft parts of dentine. They do not lie in direct contact with the hard matrix, but are invested by sheaths, the dentinal sheaths of E. Neumann which are intimately connected with the matrix. After the fibres have been removed by maceration or by incineration of the tooth the dentinal sheaths remain, and even after destruction of the matrix by boiling muriatic acid or in caustic alkalies, they constitute the only perfectly indestructible residue of the tooth."

Now this is really remarkable, and I must say that the fire used in Europe for "incineration" must be not as hot as fire is here, or the acid and alkalies not so strong as these chemicals are on this side of the Atlantic, or their sheaths of Neumann must be something of the nature of platinum or diamond. It seems indeed these sheaths are quite indestructible in the minds of the investigators there.

"The fibres easily stain with carmine. They possess a remarkable degree of etensibility, so that especially in young teeth the dentinal cells may be separated to a considerable distance from the dentine, without rupture of the processes which then appear like harpstrings stretched over the interval. Salter, in recently describing the fibres as tubules, (because when dry they appear to contain air vesicles and exhibit a dark central point on a section) had probably the dentinal sheaths under observation. The fibres are really completely solid and homogeneous."

We see here the same arguments and even Salter's writing did not seem to have made it possible for the writer to consider if not his dentinal fibre and sheath were the same thing. How a fibre can be completely solid and homogeneous, and stretched like harpstrings, (Waldeyer) and on rupturing let a thick fluid exude (Tomes) and be stained with carmine—all these properties united make really a remarkable object.

Beale's view on the structure of dentine we find in his lectures translated by Carus, Liepzig, Engelmann, page—— "There are few anatomical questions which have called forth a more lively controversy than the structure and formation of dentine; the latest author on this subject, Lent, describes the dentinal canals as consisting of the direct process of the odontoblast. "Die grundsubstanz des zahnbeines entsteht nicht ous dam Elfenbein zellen, sondern ist entweder eine Aussheidung dieser Zellen, oder dar Zahnpulpa ahnlich einer Interzellular substanz." (The basis substance of dentine does not originate from the ivory cells, but is either an execretion of these cells or of the pulp of the tooth, similar to an intercellular substance.) Tomes now has shown that the dentinal canals are tenated by a soft formation which can be seen in the form of solid processes protruding from the broken off ends of the dentinal canals [Koelliker says that the same appearance is produced by Neumann's sheaths.] The correctness of these observations has, however, been doubted by several observers. I have been able to verify the assertions of Tomes as regards the filling up of the dentinal canal with a soft formation and place before you a preparation in which this substance is colored red with carmine and can very plainly be seen. The tooth canals of a living tooth are never empty, they are no tubes at all, nor channels for the transmission of nutrient substances held in solution by fluids, but they contain a soft solid formation the central portion of which is in a condition of active vitality."

We do not doubt of course that the contents of the dentinal tubes can be stained by carmine, but must we on that account admit, that that which Tomes shows by muriatic acid, fracturing and stretching, be the same thing as that which Beale shows by staining? must we admit that because there is germinal matter in the canals there are no tubes there? That would be quite peculiar logic. Of course Beale shows with his method the inner portion of the contents of the canals, which is bioplasm. Tomes shows with his method the outer portion or the dentinal tube, which is certainly not the soft semi-fluid germinal matter, and although in a living tooth Tomes' and Koelliker's fibril contain the bioplasm of Beale, their arguments do not point out the same.

In Todd, Beale & Bowman's Physiology, page 353, Beale, when speaking of the structure of the bone gives his views more implicitly on some points. "The innermost layer of tissues constituting the wall of the lacunæ and of its canaliculi differs in resisting properly from that which is external, not that this tissue is developed separately from the germinal mass of the bone texture. Its greater hardness is probably due to its very slow formation as in the case of the so-called wall of the dentinal tube, which affords another instance of the same sort of artificial distinction of texture, and which has led to a similar view concerning its formation as a texture distinct from the so-called 'intertubular tissue.'"

Why Beale calls it an artificial distinction if attention is called to the difference in chemical composition between the homogeneous intertubular mass and the tubules themselves, I cannot understand. If nature uses something of the nature of leather, and something of the nature of glue in the contraction of her apparatuses, why have we not a right to recognize both? If Beale makes distinctions by the aid of carmine between formed and germinal matter, why are we forbidden to analyze the formed matter still further? And why is not the distinction which muriatic acid makes, of some value just as as well as that which carmine makes?

I suppose Beale opposes the special tubule because it tastes like the "cell wall," and because it opposes his doctrine that the portion of formed matter nearest to the bioplasm is always the latest formed. But this does by no means follow from the theory of bioplasm, *i. e.* that bioplasm always makes or precedes formed matter. Theories have to be made after facts, and not facts ignored on account of theory.

If it now be asked, how came this variety of theories concerning the nature of dentine I think it may be said, the reason is that there are only a limited number of facts, or observations taken in consideration when the theory is made and the causes for that are that the general theories of some will not allow a certain number of facts to become prominent, and again that by others, other facts are assumed which need proof and which are then in the way when they appear in reality.

For example, Beale dwells especially upon the distinction between formed matter and germinal matter, and therefore lays great stress on the facts revealed by carmine, while he ignores the special texture of the tubule because he is fighting the cell doctrine, and because the tubule would agree with a cell membrane. Others again neglect staining, and rely chiefly on the facts brought out by muriatic acid, or think that all soft tissues are destroyed and then imagine that they have destroyed all the soft uncalcified parts, and without giving proofs assume the presence of tubes and then when it comes in their way in different form from what they expected, they think it is something else.

I think the view here given in this paper on the structure will be agreeable to all the facts that have been brought out, and not only that, but in forming the conclusion almost every fact was used. There was neglected neither the staining nor muriatic acid, nor stretching the tubules or fibres, nor the dead tooth nor the living and growing tooth, neither longitudinal nor cross-sections. This view of structure of dentine it will be seen has its support further in the structure of bone, tendons, cornea, whartons, jelly and others. For here we have also the yellow elastic substance either in membranes or in fibres, or threads, together with a homogeneous substance between them which differs also in the different tissues being like glue, for example, in tendon and like mucus in the fibrous tissue of umbilical cord. And as to the direct production of the yellow elastic we have an unmistakeable proof for that in the development of the ligamentum nucae where their is no other texture formed but the yellow elastic fibre and where we can see that these are a direct product of the germinal matter which we find connected with them.—*Dental Register.*

